# Motor skill delays in pre-school children with leukemia one year after treatment: Hematopoietic stem cell transplantation therapy as an important risk factor

**DOI:** 10.1371/journal.pone.0186787

**Published:** 2017-10-24

**Authors:** Livia Taverna, Marta Tremolada, Sabrina Bonichini, Barbara Tosetto, Giuseppe Basso, Chiara Messina, Marta Pillon

**Affiliations:** 1 Faculty of Education, Free University of Bolzano-Bozen, Faculty of Education, Brixen, Italy; 2 Department of Developmental Psychology and Socialization, Faculty of Psychology, University of Padua, Padua, Italy; 3 Medical School for Health Professions “Claudiana”, Bolzano, Italy; 4 Department of Child and Woman Health, Oncology Hematology Division, University-Hospital of Padua, Padua, Italy; German Cancer Research Center (DKFZ), GERMANY

## Abstract

CNS-directed therapies for the treatment of leukemia can adversely affect the acquisition of new skills, such as reading/writing and math. Two years after the end of treatments, children show gross and fine motor skill delays that may persist even when patients are considered healed. The goal of the present study was to assess motor skills difficulties in pre-school children with leukemia one year after treatment. Particular attention has been paid to those patients who had undergone Hematopoietic Stem Cell Transplantation (HSCT) and to the relationship between motor delays and age bands. Participants were 60 children (median age of 5; inter quartile range: 3.07–5.76), including 31 females and 29 males, 91.7% of them were affected by acute lymphoblastic leukemia (ALL), and 8.3% by acute myeloid leukemia (AML). Five children had undergone HCST. Parents were interviewed by Vineland Adaptive Behavior Scales (VABS) on children’s motor skills and filled in the Italian Temperament Questionnaire (QUIT). VABS’s total scores were converted into equivalent mental age scores (EMA). A score difference of at least three months between current age and equivalent mental age was considered a developmental delay. Non-parametric analyses were run to understand if HSCT treatment and a specific age band influence children’s motor skills. Significant delays were found in global motor skills (56.7%) as well as in fine and gross motor domains. Mann Whitney U tests showed that children with HSCT were reported to have lower gross motor mean ranks (U = 62; p = 0.004; *Mean rank* = 15.40) than peers without HSCT (*Mean rank* = 31.87) and lower mean rank values on motor temperament scale (U = 9; p = 0.003; HSCT *Mean rank =* 4.75 versus no HSCT *Mean rank* = 27.81). Kruskal Wallis’ tests identified the high risk treatment showing that HSCT experience negatively impacted the motor skills and temperamental motor activity of pre-school children one year after the diagnosis of leukemia.

## Introduction

In the last 15 years, malignant neoplasms in the age group 0–14 years have shown a dramatic increase (+12%) in 5-year survival, from 70% in 1988–1993 to 82% for cases diagnosed in 2003–2008 [1]. More attention has been paid to understanding the impact of treatments and interventions on children’s development, particularly with respect to late effects on academic, physical, and social functioning, essential for optimization of long-term outcomes [2]. However, research on children with cancer has mainly focused on childhood cancer survivors [3], and less is known about skills or abilities that enable the pediatric patient to function adequately during cancer treatments [4]. Adaptive functioning reflects application of the children’s abilities in daily life at an age appropriate level and is strictly associated with the general concept of quality of life.

In this study, we focus on the development of motor skills in pre-school children with leukemia. Basic motor competencies are acquired early in life, reflecting maturation in interaction with formal or incidental learning [5, 6]. Chemotherapy could induce delayed skeletal muscle dysfunction in survivors of acute lymphoblastic leukemia (ALL) [7]. In addition, pain and fatigue limit physical functioning [8], with gross and fine motor problems evident in leukemia survivors even two years after cessation of treatment [9–12] and with significant persistent visual-motor deficits, especially in girls younger at the diagnosis and if the time since the end of treatment is short [13]. These basic processing skills are necessary for the development of higher-level cognitive abilities, including non-verbal intelligence and academic achievement, particularly in arithmetic and written language [14].

The cognitive impairments in children with leukemia can be related to Hematopoietic Stem Cell Transplantation (HSCT) [15], with IQ and adaptive behavior scores dropping significantly during the first year and not changing in successive follow-ups (from one to three years). A study on childhood HSCT survivors [16] found that academic ability was rated significantly lower than in the normative group but higher than in the learning disability normative group, while cognitive ability (IQ) remained stable two years after HSCT. A longitudinal study on progressive declines in neurocognitive function among survivors of HSCT [17] showed declines in visual motor skills and memory test scores within the first year post-HSCT. By three years post-HSCT, there was improvement in visual motor development scores and memory scores, but there were new deficits in verbal skills. Two years post-HSCT, performance IQ and processing speed were above the norm values, whereas arithmetic and motor scores were below [18]. A recent review [19] found that muscle strength and balance seem to be impaired in varying degrees in children with cancer during and off treatment as well as several years after cessation of treatment, in accordance with current discussions about long-term survivors being at risk for muscle weakness, poor fitness, and frail health [20]. The child’s temperament could also be identified as an important stable factor that impacts adaptation to the illness and quality of life [21], even if it has never been considered as a dependent variable influenced by the cancer parameters.

Based on the literature discussed above, the current study’s aims and hypothesis are as follows:

1. Preschool children with leukemia could show just during the maintenance phase significant motor skills delays, in both gross and fine motor skills [14, 19].
2. We wanted to compare the motor activities temperament score of these healed children with Italian norms to see if they were lower.
3. Days of hospitalization and HSCT treatment could influence negatively motor abilities [16, 18].
4. We also aimed to verify if the motor skill deficits were more present in pre-school children with leukemia at a particular age, a topic not yet taken into consideration.

## Materials and methods

### Participants

Participants included 60 children with a median age of 5 (inter quartile range: 3.07–5.76), including 31 females and 29 males. The great majority of participants were affected by acute lymphoblastic leukemia (ALL = 91.7%), whereas a few of them were diagnosed as having acute myeloid leukemia (AML = 8.3%). Childhood cancer patients reported a median value of 40 days of hospitalization (inter quartile range: 34–62). Twelve children belonged to the standard risk protocol, 41 to the intermediate protocol, and 7 to the high-risk protocol; 5 underwent HCST.

All parents were Western European descent with a median age of 37 (inter quartile range: 34–41) and a median of 13 years of schooling (inter quartile range: 8–13). Parents’ incomes were mostly average (51.7%), followed by high (28.3%) and low (20%) for Italian norms, but above poverty. The median of job hours/weekly was 35 (inter quartile range: 5.25–35). Some parents were temporarily relieved from their work or were housewives (40%), some worked full time (35%) and part-time (20%), and a percentage (5%) had lost their job. The number of children for each family was mostly two (N = 38), followed by one (N = 17) and three (N = 5). Table 1 shows the children and families characteristics.

**Table 1:** Socio-demographic and clinical characteristics of pediatric patients.

		*Frequencies*	%	*Median*	*DS and inter-quartile*
***Gender***	Male	31	51.67	5	inter quartile range: 3.07–5.76
	Female	29	48.33		
	Total	60	100		
***Present age***	2–3 years	12		19.28	2.96
	4 years	11			
	5 years	18			
	6 years	19			
***Parent’s education (schooling years)***	13 years of schooling			13	inter quartile range: 8–13
***N° of siblings***	No sibling	27	27.0		
	1 sibling	17	56.0		
	2 siblings	38	13.0		
	3 siblings	5	4.0		
***Employment***	Job leave/housewife	24	40		
	Abandonment / loss of work	3	5		
	Part time	12	20		
	Full time	21	35		
***Job hours/weekly***				35	inter quartile range: 5.25–35
***Economic situation perceived***	Low	12	20		
	Medium	31	51.7		
	High	17	20		
***Home situation***	Rent home	12	20		
	Home ownership with mortgage	19	31.7		
	Home ownership without mortgage	24	40		
	Other	4	6.7		
	Not reported	1	1.7		
***Diagnosis***	Acute Lymphoblastic Leukemia	55	91.7		
	Acute Myeloid Leukemia	5	8.3		
***HCST***	No	55	91.67		
	Yes	5	8.33		
***Risk band***	Standard Risk (SR)	12	20		
	Medium Risk (MR)	41	68.3		
	High Risk (HR)	7	11.7		
***Days of hospitalization***				40	inter quartile range: 34–62

### Procedure and measures

Ethical approval was obtained from the University Hospital of Padua Committee. The parents were contacted by a clinical psychologist during the first hospitalization of the children, about one week after the diagnosis. The project aims were explained, and written informed consent was asked for. Informal contacts with the participants were kept up on a daily basis to provide support and motivation for the project. The participants were informed that they were free to drop out at any moment of the study. Each family was contacted again 12 months later, when the Vineland Adaptive Behavior Scales (VABS) [22, 23] and the Italian Temperament Questionnaire (QUIT) [24] were administered to the parents, with particular attention to motor skills. Medical and socio-demographic information was also collected.

From the initial sample, we took into consideration only the pre-school-age group (23–74 months), which included 75 children at that point. Of these, 15 exited the study for several reasons: deceased or in a terminal condition (N = 9) or changed the hospital or decided to drop off (N = 6). The response rate was 92%, excluding the deceased patients. The assessments were carried out at the Day Hospital or in the library of the clinic.

The VABS includes a psychometrically validated parent interview administered by a trained psychological examiner that provides norm-referenced scores on a range of adaptive behaviors at developmental levels from birth through adulthood throughout several domains (personal and social). It has 540 items organized around four adaptive behavior domains: communication, daily living skills, socialization, and motor skills. Each domain includes several subdomains. The motor scale, used in this study, measures gross and fine motor abilities. Each item is rated “2” (behavior is usually or habitually performed), “1” (sometimes or partly performed), or “0” (never performed). In addition, there is a code (“N”) for cases when the child has never had the opportunity to perform the activity, as well as a code (“DK”) to use when the caregiver does not know if the child performed the activity.

The reliability and validity of the VABS and their psychometric properties have solidified this measure as one of the most widely used assessments of adaptive behaviour. Domain and subdomain raw scores are obtained by summing up the numerical values of the responses. Using tables in the manual, the raw scores can be converted into standard scores (with a mean of 100 and standard deviation of 15), percentile ranks, stanines, and age equivalents. The sum of the domain standard scores is used to obtain the composite standard score. A table is then used to obtain the stanines and percentile rankings for the composite from the standard scores. The age equivalents for the composite score can be either the mean or median of the domain age equivalents. In this study, the total score for VABS motor skill subscales was converted into the equivalent mental age score; the score difference of at least three months between the current age and the equivalent mental age was considered a developmental delay.

The QUIT measures temperament in children aged from 1 month to 11 years. The QUIT has different versions according to age ranges (1–12 months; 12–36 months; 3–6 years; 7–11 years) and consists of different subscales that measure attention, motor activity, social interaction, and positive and negative emotions. Each questionnaire is composed of 60 items to be filled in by the parent. Completion time requires approximately 15 minutes. The QUIT, which was administered to parents of 775 children distributed over two age groups considered here (1–12 months and 13–36 months), demonstrated good internal consistency (Cronbach alphas ranging from .59 to .83).

Each parent filled in a socio-demographic questionnaire with inquiries into their highest year of schooling, education, perceived economic situation, type of home situation, relationship status, and type of employment.

The oncologists who followed the patients extracted the necessary data from the patients’ medical records. The medical data extrapolated from the records included the date of diagnosis, type of leukemia, therapy protocol involved (SR, MR, HR), age at diagnosis, and HSCT (yes/no).

### Statistical analyses plan

We ran descriptive statistics to show the child’s adaptive behavior scores one year after treatment, specifically the scores related to motor skills and their subscales (gross and fine). The data were checked for normality adopting the Kolmagorov-Smirnov and Shapiro-Wilk tests. The distribution of the data was not normal, so we decided to use non-parametric statistics.

Preliminary Spearman’s ranks correlations were run to identify the possible significant associations between the variables. Results have been considered significant with a p value ≤ 0.05. To control the familywise error rate we adopted the Bonferroni post-hoc adjustment for multiple comparison, accepting only correlation reaching the critical value of p ≤ 0.007 since the number of comparisons carried out was 7 (p = 0.05/7). These preliminary observations allowed us to identify the most critical variables to use in the further analyses.

A series of four Mann Whitney U tests were run to determine if there were differences in the VABS’s and QUIT’s motor scores between children treated with HSCT and those without HSCT. In this case motor scores were the dependent variables and the independent variable was the treatment group (HSCT presence vs absence).

A series of Kruskal-Wallis χ^2^ tests were run with the risk band (Standard risk, SR; Medium risk, MR; and High risk, HR) as independent variable and the VABS motor scores as dependent variables. The risk band reflects the intensity of the treatment adopted for each type of leukemia following an accredited medical international protocol. Independent variables involved in these analyses were ordinal since childhood cancer differ with respect to treatment severity (SR, MR, HR), as well as type of therapy (HSCT is considered a more aggressive treatment compared to others).

A series of Kruskal-Wallis χ^2^ tests evaluated if the negative difference (age in months) between Chronological Age (CA) and Equivalent Mental Age (EMA) respectively in Global, Gross and Fine motor skills could be influenced by children’s belonging to a specific age group (2–3 years old, 4 years old, 5 years old, 6 years old). Bonferroni post hoc test was not needed since the reduced number of comparisons.

The Mann-Whitney U and the Kruskal Wallis χ^2^ tests are rank-based nonparametric tests used to determine if two/three groups differ on a continuous or ordinal dependent variable.

All data were analyzed using SPSS version 22 (SPSS Inc., Chicago, IL, USA).

## Results

**Fig 1:**
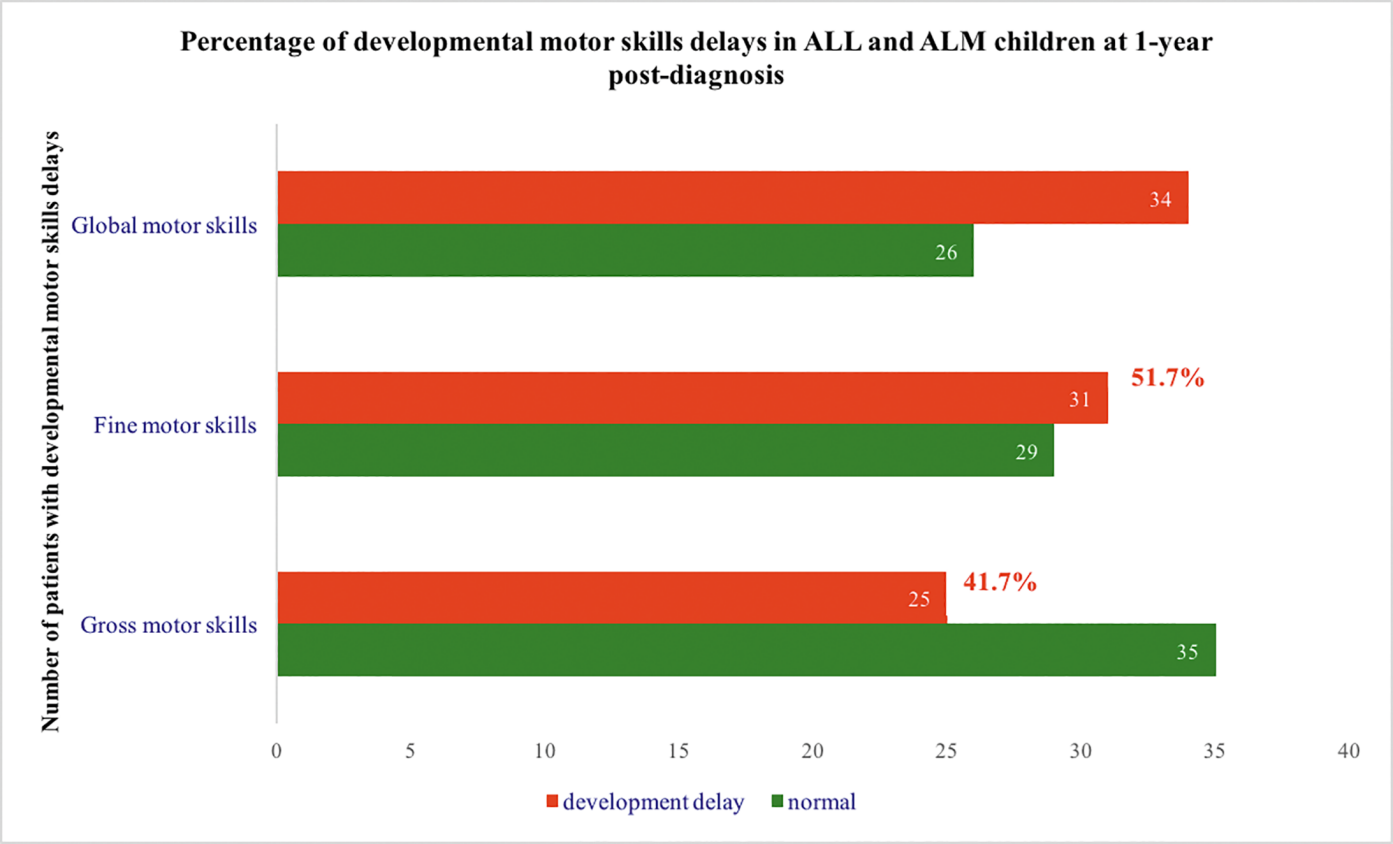
Percentage of developmental motor skills delays in ALL and AML children at 1-year post-diagnosis.

Descriptive statistics of motor skill delays are reported in Fig 1. A score difference of at least three months between current age and equivalent mental age was considered a developmental delay. Significant delays were found in global motor skills (56.7%; N = 34), as well as in fine (51.7%; N = 31) and gross motor skills (41.7%; N = 25).

The temperament motor scale showed a mean of 4.72 (SD = 0.80; range 1.36–5.50), principally concentrated in a high level according to the norms (51%), followed by 38.3% placed in a medium level and 10.6% at a low level.

Spearman’s ranks correlations showed associations between medical and socio-demographic factors, motor abilities, and temperament scores. Global motor abilities were positively associated with the diagnosis of ALL (r_p_ = -0.33; p = 0.01), while the absence of HSCT was positively associated with gross motor subscale (r_p_ = -0.2; p = 0.04) and with QUIT motor scale (r_p_ = -0.42; p = 0.002). The diagnosis of ALL was also statistically significant related with children’s performance on VABS’ fine motor subscale (r_p_ = -0.32; p = 0.013) and QUIT motor scale (r_p_ = -0.32; p = 0.021). Adopting post hoc Bonferroni correction, we considered a significant association only with correlations showing a p-value ≤ 0.006 (Table 2).

**Table 2:** Spearman’s correlations between medical and socio-demographic variables and children’s motor skills.

	QUIT Motor Scale	VABS Global Motor Skills	VABS Gross Motor Skills	VABS Fine Motor Skills
Child’s gender	r_p_ = -0.13	r_p_ = -0.14	r_p_ = -0.10	r_p_ = -0.11
	p = .34	p = .27	p = .44	p = .38
Mother’s age	r_p_ = 0.065	r_p_ = 0.28	r_p_ = 0.17	r_p_ = 0.31
	p = .66	p = .03	p = .19	p = .02
Mean job hours/week	r_p_ = -0.98	r_p_ = -0.26	r_p_ = -0.31	r_p_ = -0.20
	p = .49	p = .044	p = .016	p = .12
Parent’s years of schooling	r_p_ = -0.24	r_p_ = -0.062	r_p_ = -0.078	r_p_ = 0.01
	p = .07	p = .64	p = .555	p = .93
Days of hospitalization	r_p_ = -0.07	r_p_ = -0.093	r_p_ = -0.14	r_p_ = -0.04
	p = .62	p = .48	p = .28	p = .76
HSCT (yes/no)	**r**_**p**_ = -0.42*	r_p_ = -0.22	**r**_**p**_ = -0.26*	r_p_ = -0.23
	**p = .002**	p = .094	**p = .004**	p = .09
QUIT Motor scale		**r**_**p**_ = 0.32*	**r**_**p**_ = 0.39*	r_p_ = 0.23
		**p = .002**	**p = .004**	p = .09

Note

*correlation significant at p ≤0.007 adopting the Bonferroni correction for the 7 comparisons

Taking into consideration the significant associations, we then ran a series of Mann Whitney U tests to see if Equivalent mental age (EMA) on motor skills (VABS) and temperament (QUIT) scores significantly changed along the presence/absence of HSCT. Distributions of EMA on motor scores for children with HSCT and without HSCT were not similar, as assessed by visual inspection. VABS’s EMA on motor scores for males (mean rank = 23.25) and females (mean rank = 17.75) were not statistically significantly different, *U* = 145, *z* = -1.488, *p* = 0.137.

**Fig 2:**
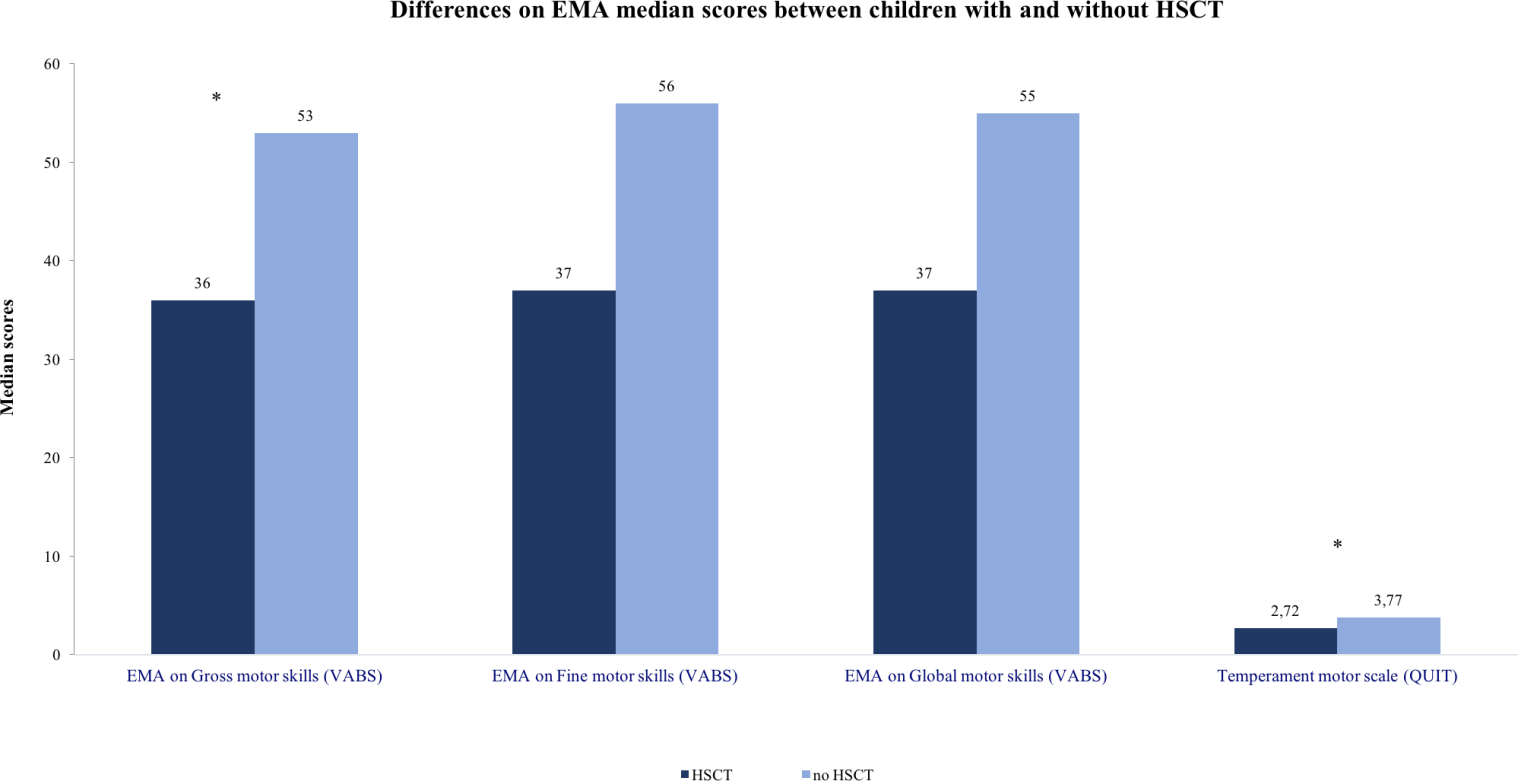
Differences on EMA median scores between children with and without HSCT. EMA = Equivalent Mental Age.

Fig 2 shows the results of this analysis. The presence of HSCT negatively influenced the motor temperament (U = 9; p = 0.001; Median motor temperament in children with HSCT = 2.72 versus 3.77 in children without HSCT) and the EMA on gross motor skills of children (U = 62; p = 0.04; Median gross motor skills in children with HSCT = 36 versus 53 in children without HSCT).

**Fig 3:**
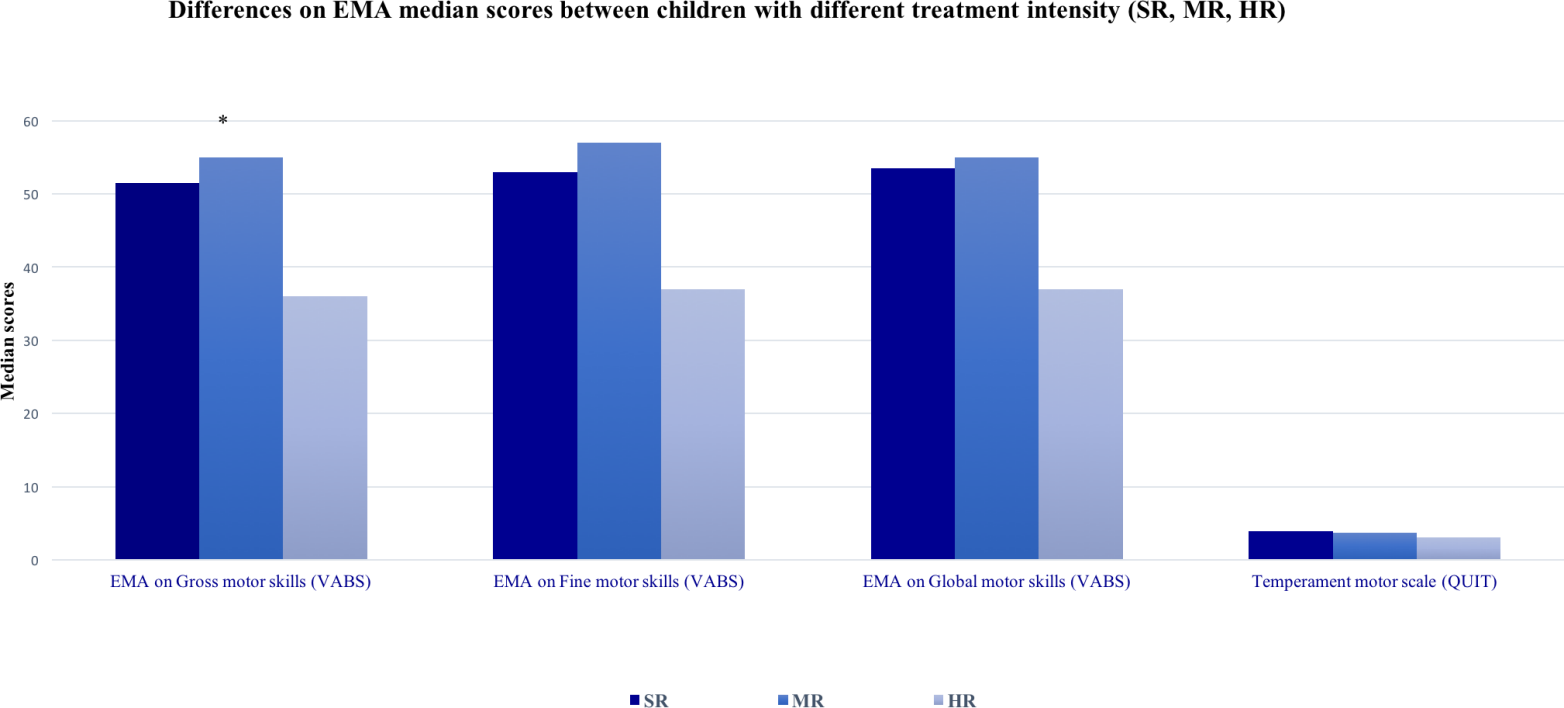
Differences on EMA median scores between children with different treatment intensity (SR, MR, HR). EMA = Equivalent Mental Age; SR = Standard Risk; MR = Medium Risk; HR = High Risk.

A series of Kruskal-Wallis χ^2^ tests were run with the risk band (Standard risk, SR; Medium risk, MR; and High risk, HR) as independent variable and the VABS motor scores as dependent variables. Fig 3 shows the results of these analyses. The HR treatment (Median = 36) was significantly associated with children’s lower EMA on gross motor skills (χ^2^ = 5.81; df = 2; p = 0.05) if compared to EMA of the SR treatment group (Median = 55) and those of the MR treatment intensity (Median = 51.5).

**Fig 4:**
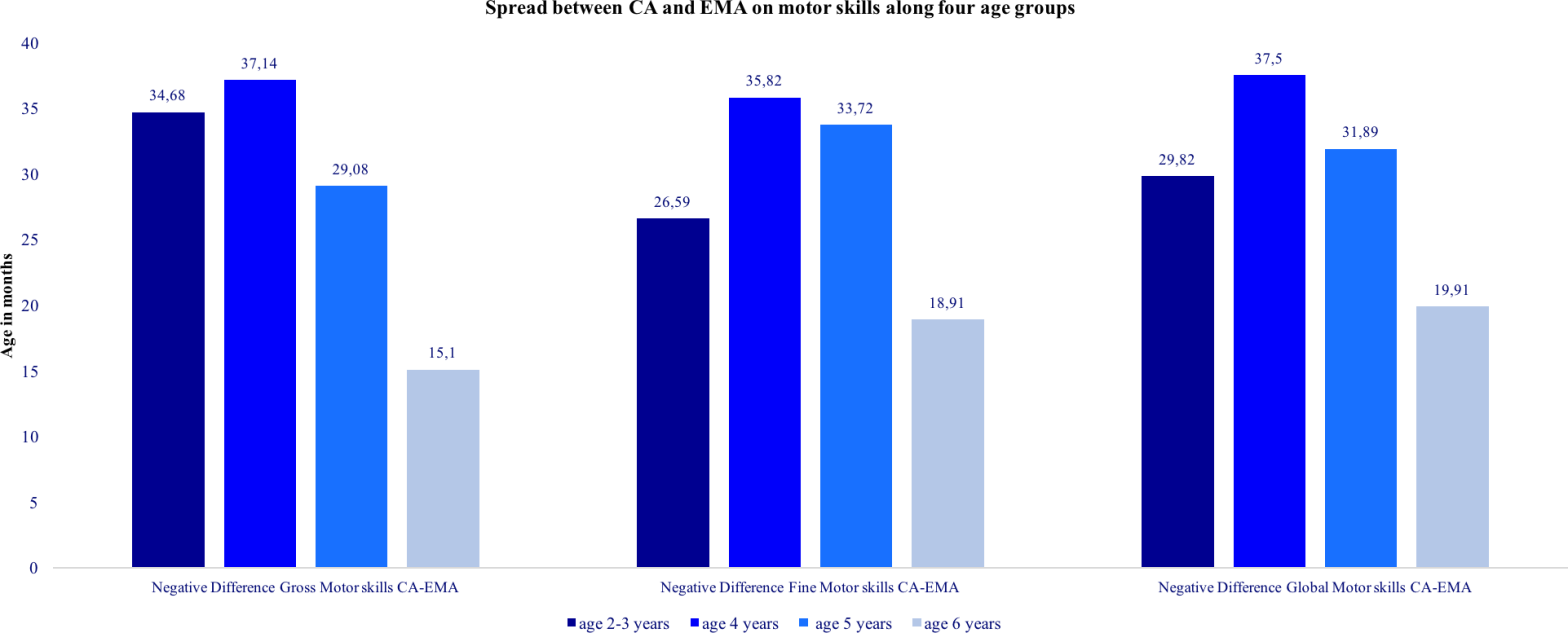
Spread between CA and EMA on motor skills along four age groups. Note: KRUSKAL WALLIS with Age range (4 levels) as independent factor and Negative mean ranks difference between Chronic Age (CA) and Equivalent Mental Age (EMA) respectively in Global, Gross and Fine motor skills as dependent variables.

We also ran a series of Kruskal-Wallis χ^2^ tests (Fig 4) to evaluate if the negative difference between Chronological Age (CA) and Equivalent Mental Age (EMA) respectively in Global, Gross and Fine motor skills could be higher if children belong to a specific age group (2–3 years old, 4 years old, 5 years old, 6 years old). The different mean ranks distribution along age groups resulted of significance both in fine motor skills (χ^2^ = 9.76; df = 3; p = 0.02), in the gross motor scale (χ^2^ = 15.32; df = 3; p = 0.002) and for the global motor skills (χ^2^ = 8.57; df = 3; p = 0.03). Examining the mean ranks, the negative difference between chronological age and equivalent mental age in Global motor skills and in its subscales Fine and Gross ones was lower in children aged 6, than those aged 2–3, 4 or 5 years old. Fine motor delays affected above all 4–5 years old-children.

## Discussion

In this study, we documented significant impairments in children in both gross (41.7%) and fine motor skills (51.7%) already after one year of treatments. Developmental delays were defined with a score difference of at least three months between current age and equivalent mental age on the VABS assessment. Consistent with the main findings reported in other studies our results confirm a clinical concern for expected motor development of pre-school children still after one year of treatment and in the maintenance phase [25, 14]. Despite the fact that a third part assessment tool was used to determine leukemia children’s actual motor skills, it is necessary to notice that VABS interview does not ask parents to express attitudes or impressions about their children, but to report manifest and concrete behaviors that they are likely to observe [6]. Furthermore, reliability studies on this measure have shown good interrater correlation coefficients between informants with Cronbach’s alpha greater than .85. In this way reported differences between leukemia children and the normative sample reflect true differences in motor functioning and document a gap between what normally developing children are expected to be able to do at this age and the developmental achievements that cancer patients effectively master. Furthermore the VABS interview has been used in Italy previously to assess normal child behavior [26, 27] and children with leukemia or other tumors [28, 29].

Other studies providing information about the impact of chemotherapic agents on motor skill development in childhood cancer survivors adopted different research methods including direct measures of motor performance. Recent findings report up to 33% of ALL patients showing impairments on formal assessment of the motor domain after completion of therapies [12, 19]. De Luca and colleagues [12], for example, examined the motor performances of thirty-seven leukemia patients at three time-off-treatments on the eight tasks of the Movement Assessment Battery for Children, version 2 (MABC-2). More than a fifth of participants (21.6%) displayed fine motor impairments, and approximately one third (27%) scored below the 15^th^ percentile on the global motor domain if compared with the normative population. Fine motor delays were more prevalent two years after treatments in children aged 7 to 9 years even though time-off-treatments did not affect children’s performances. While impairment rates did not increase significantly with greater the time from completion of therapy, authors suggest that this emerging “time effects” reflects the difficulty to cope with the increasing task demands required at this age, rather than to be a late-treatment outcome. Nevertheless fine motor skills appear to be significantly reduced compared to standardized motor norms raising clinical concern for long term difficulties that need to be tracked and monitored to support children’s quality of life.

In contrast with the illness’s limitations, the HSCT children in our study reported a high score on the QUIT motor scale compared with the Italian norms (51%), showing a temperamental physical activity predisposition. This gap between motor temperamental nature and physical constraints may elicit frustration, which in turn could result in negative effects on the development of these children (scarce medical compliance, depressive mood, poor motivation towards any school activity, etc.). These two measures on the motor domain were also associated with each other, showing how temperamental factors and effective motor performance are linked to each other.

Previous research investigated later effects of chemotherapy on overall physical conditions in ALL survivors documenting higher health risks for these patients if compared with healthy peers. Muscle weakness, poor fitness, pain, and fatigue limit physical function [8], and delayed skeletal muscle dysfunction [7] of childhood cancer survivors have been reported also one year after treatments. In addition, lower hand grip strength has been associated with higher cumulative doses of glucocorticoids in survivors of childhood cancer, even ten years after diagnosis [20]. Adequate hand strength is very important and is required for performance of various fine motor tasks which are characterized by small muscle movements that usually occur in the fingers [30]. Fine motor abilities include, among others, coloring, drawing, writing and graphomotor skills whose quality is related to the strength and control of finger muscles. Accordingly to these studies, our findings provide evidence that preschool children affected by cancer early in life, show impairments on fine and gross motor skills already one year after treatments, with longer hospitalizations and the HSCT treatment as negatively associated with their performances on these domains. This finding might be taken as an empirical support to the fact that the duration and the intensity of treatments hampers children to develop and refine a variety of motor skills that are usually practiced in the daily life and are necessary also for later academic success. Accordingly to our results, special attention should be paid to patients that underwent a high intensity of leukemia treatments that have been found to be more at risk in their motor functioning, both in fine and in gross motor skills.

The results of this study showed how children who had undergone HSCT obtained significantly lower scores in equivalent mental age in the gross motor domain and on the motor temperament scale. The equivalent mental age is just adjusted for children’s age, so that the experience of HSCT is the unique risk variable identified. This difficult experience may have influenced not only the motor physical functions but also the temperamental disposition of the children, which dampens their motor attitudes.

The negative difference between chronological age and equivalent mental age in gross motor skills was higher in children aged 2–3 and 4 years than those of the other ages (5 and 6 years). Also, fine motor skill delays were distributed differently among the age groups and were especially concentrated in children aged 4 and 5 than those aged 2–3 or 6 years old. Presumably, children with motor delays in early childhood might experience greater difficulties in the learning phase of handwriting, since fine motor skills and graphomotor skills are required to master tasks like letter formation, size and spacing between writing traces. Berninger and Fuller [31], for example, suggest that handwriting may be particularly challenging for students who lack on foundational skills in writing. The transition from kindergarten to first grade is an important period to develop and practice fine motor skills. In an observational study on 4 year-olds children Marr and colleagues [32] reported that kindergarteners spent approximately half of their school day engaged in fine motor tasks (range of 36%-66%). About the 20% of these activities were paper and pencil activities for either play or learning (writing or coloring with a pencil, crayon, or marker, or painting with a paintbrush). Two years later, children in second grade were found to spend as much as 30–60% of their day participating in an activity that required fine motor skills, of which 85% involved paper and pencil tasks [33]. On the basis of above mentioned studies it could be hypothesized that children beginning the elementary school with important delays in fine and gross motor domains could be more at risk for academic achievement. Moreover, longer hospitalizations and necessary treatments like HSCT contribute to limit the discovering of motor functioning at this age stage forcing the young patients to stay in bed, and to avoid social and physical contacts due to their immunocompromised status. Therefore further longitudinal research is needed to understand how impairments on fine and gross motor domains in patients survived to childhood cancer may affect their later academic achievements.

Accordingly to our findings, motor skills in children with leukemia should be assessed already during treatments, because these basic foundational skills are necessary to the development of higher-level cognitive abilities, including non-verbal intelligence and academic achievement, particularly in arithmetic and written language [14]. Visual–motor integration has been found to be one of the most significant predictors of handwriting performance [34–36]. Pain and fatigue may impact handwriting proficiency, leading to less legible written texts. Legibility is one non-content factor that influences readers’ judgements about the quality of ideas in a written text. Children experiencing difficulties with handwriting might be judged as poor handwriters, develop a mindset that they cannot write [37], and avoid this academic requirement whenever possible [38–39].

Screening children with impairments due to HSCT and early intervention on their fine and gross motor development could improve their school adaptation. It is generally assumed that fine motor skills are critical to handwriting development because they involve the strength and control of finger muscles. Furthermore, graphomotor skills imply an ordered sequence of motor movements, finger dexterity, and wrist extension [40]. These are related to important daily skills, such as drawing and writing, as precursors of academic achievement [41]. Future research could adopt a longitudinal design and re-evaluate these children in their motor abilities along several steps in order to understand the possible changes over time and the possible presence of other academic achievement and physical functioning delays.

Children who had undergone HSCT and with a high risk protocol treatment should be monitored more specifically, and specific exercise programs should be set up. For example, a pilot study demonstrated that home-exercise intervention during ALL maintenance therapy was feasible and had promise for efficacy [42]. Also, specific occupational therapy should be proposed at the hospital, at home, and at school [43].

This study has some limits deriving from the fact that it has a one time-point design, and other longitudinal assessments should be added to understand the developmental trajectory of these children. The HCST children involved in this study are reduced in number, so our findings may not be generalizable. Our results were moderate significant (p ≤ 0.05) and should be taken with caution as preliminary findings. However, this study also has some strengths. It assesses only pre-school children with leukemia, a specific population in which motor abilities assume a key role in their future development and in which the duration of therapies is longer. The study uses in-depth and structured interviews with parents regarding their children’s adaptive behaviors, which allowed the comparisons with norms and gave us an indication of the amount of delay. Finally, this study takes into consideration both observational adaptive behavior aspects and temperamental factors in motor abilities, showing how motor attitudes could be associated with motor functioning.

## Supporting information

S1 Dataset (XLSX)
